# 

**Published:** 1855-04

**Authors:** 


### RECEIPTS FROM FEB. 27 UP TO MARCH 27, 1855.

ILLINOIS.

| | | | |
|---|---|---|---|
| Dr R F Henry | $3 00 | Dr E E Webster | $2 00 |
| O M Long | 3 00 | M S Irvin | 2 00 |
| R S Goodwin | 2 00 | J Blount | 2 00 |
| H Shoemaker | 2 00 | A Young | 2 00 |
| H C Morey | 2 00 | J Bowen | 2 00 |
| G W Kittell | 2 00 | S M Reynolds | 5 00 |
| W E Clarke | 1 00 | J L Gray | 2 00 |
| P A Allaire | 2 00 | H Cutler | 6 00 |
| S O Long | 2 00 | | |

INDIANA

| | | | |
|---|---|---|---|
| Dr J G Thompson | 4 00 | Dr T P Seller | 2 00 |
| | | J W Clark | 2 00 |

| | | | |
|---|---|---|---|
| Dr J Lewis | $2 00 | Drs T A and J G Graham | $2 00 |
| W Mathews | 2 00 | | |
| H Bonnell | 2 00 | | |

| | |
|---|---|
| Dr James P Hall, Michigan | $2 00 |
| John W Hall, ,, | 2 00 |
| Bradley Noys, Wisconsin | 6 00 |
| R W Earl ,, | 2 00 |
| H Fisk, Iowa | 2 00 |
| J H Griffith, Iowa. | 2 00 |
| J G Goodpasture, Tennesseo, | 2 00 |
| Thomas P Bond, Ohio, | 1 00 |

